# Proposal of a New Hybrid Breeding Method Based on Genotyping, Inter-Pollination, Phenotyping and Paternity Testing of Selected Elite F_1_ Hybrids

**DOI:** 10.3389/fpls.2019.01111

**Published:** 2019-09-18

**Authors:** Katarina Rudolf-Pilih, Marko Petkovšek, Jernej Jakše, Nataša Štajner, Jana Murovec, Borut Bohanec

**Affiliations:** ^1^Biotechnical Faculty, University of Ljubljana, Ljubljana, Slovenia; ^2^Faculty of Mathematics and Physics, University of Ljubljana, Ljubljana, Slovenia

**Keywords:** F_1_ hybrid breeding, doubled haploids, testing of combining ability, paternity determination, simple sequence repeats markers

## Abstract

Testing inbred lines for their combining ability is, due to high numbers of line to line testing needed for determination of hybrid performance, the most limiting factor in the F_1_ hybrid breeding procedure. We propose a novel method of F_1_ hybrid breeding that enables evaluation of large number of line to line crosses for their hybrid performance. Inbred lines (preferably doubled haploid - DH) are produced from heterozygous populations, genotyped and maintained. A group of lines is inter-pollinated randomly and their progeny examined. To identify elite F_1_ hybrids, these individual plants are selected by their superior phenotypic characteristics. Finally using paternity testing only of selected hybrids, the origin of paternal lines is revealed. To predict the number of F_1_ offspring needed in relation to the number of inbred lines being inter-pollinated, a mathematical formula was developed. For instance, using this formula for the inter-pollination of 60 distinct lines, the probability of obtaining all descendants of paternal-parent lines in a maternal-parent row represented at least once is achieved with 420 F_1_ plants in a row (p = 0.95). In a practical experiment with white cabbage, DH lines were produced using microspore culture; plants were grown to maturity and genotyped at eight polymorphic SSR loci. Two groups of lines (36 and 33 lines per group) were inter-pollinated by two methods, either using cage pollination with bumblebees or using open pollination in isolated field. A total of 9,858 F_1_ plants were planted and based on their phenotypic characteristics 213 were selected as elite phenotypes. 99 of them were genetically diverse and 5 of them were selected as super elite. Selected plants were analysed by the same SSR markers and the paternal origin of selected F_1_ plants was determined. Out of 213 selected elite plants 48 were reciprocals thus exhibiting power of selection based on single plant. We demonstrate that this new approach to hybrid development is efficient in white cabbage and we propose breeders to test it in various vegetable and crop species. Moreover, some other aspects of the proposed technique need to be tested and verified both for practical and economic criteria.

## Introduction

Hybrid breeding methods have long been used to efficiently produce varieties with superior performance and are today considered the optimal choice due to the expressed heterotic effect, uniformity, and fast trait combination and as a form of intellectual property protection. Although causal factors and genetic mechanisms of heterosis remain not completely understood (Berlan, 2018; Miyaji and Fujimoto, 2018; Govindaraju, 2019) hybrid breeding is now applied in majority of crops.

Several textbooks, such as those by Fehr (1993), Simmonds and Smartt (1999) or Acquaah (2012), and research papers (Nishio and Kitashiba, 2017; Dimitirijevic and Horn, 2018) describe methods for breeding hybrid cultivars of various species. To produce an F_1_ hybrid variety, several putative parental lines obtained from heterozygous sources are first made homozygous by several generations of inbreeding from a genetically heterogeneous genepool. Recently, haploid induction followed by chromosome doubling has frequently replaced selfing and is now recognised as the most convenient method to produce inbred lines (Murovec and Bohanec, 2012). Alternatively, by accelerated selfing to up to six generations per year, inbreeding can be achieved by the process termed as “speed breeding” (Watson et al., 2018). This method does not lead to complete homozygosity but has so far been developed for several predominantly cereal and legume species. Once inbred lines are created, breeders attempt to recognise which crossing combination of lines give superior offspring, a well-known effect termed heterosis.

Although genetic mechanisms that cause heterosis are still not completely understood (Chen, 2013), several methods used by breeders are well established and are focused on the identification of lines with optimal “combining ability” (Acquaah, 2012). Combining ability is the phenomenon that comes forth only when some inbred lines are crossed with each other, complementing each other in desired traits. Plant breeders often tend to produce a large number of inbred lines in a breeding cycle, one thousand or more being very common. It is well known that it is impossible to perform all cross combinations, for practical reasons. To perform all possible combinations without reciprocals, n(n-1)/2 crosses are required (n being the number of inbred lines). Testing the combining ability of each of a hundred lines would require 4,950 non-reciprocal crosses. Therefore, in a standard F_1_ breeding scheme, the first step is testing for “general combining ability”, meaning testing of a large number of lines with one or more “tester lines”. Based on progeny performance, the best are chosen for “specific combining ability (SCA)” testing, i.e., line-to-line crossing in a single or reciprocal way. Particularly in vegetable breeding, F_1_ hybrid breeding protocols tend to exclude testing for general combining ability; therefore, the performance of a limited number of previously determined superior seed lines is predominantly tested by various pollen parents. Such search for optimal combining ability in relation to metabolites was presented in white cabbage by Singh et. (2018).

Testing inbred lines for their combining ability is the most limiting factor in the F_1_ hybrid breeding procedure. Therefore, some attempts were made to overcome this bottleneck. An alternative to testing for combining ability termed “reverse breeding” was proposed by Dirks et al. (2009) and elaborated by Wijnker et al. (2012). In their method, superior individual heterozygous plants are first identified within a population, then due to achiasmatic chromosomes, formed by the insertion of genes preventing crossing over, gametes with limited recombination frequency are formed. The following steps aim to create an identical F_1_ hybrid as the original heterozygote required haploid induction and the monitoring of individual chromosomes by molecular markers. The method has several limitations: it can only be used in species with small chromosome numbers and the transgenic status of intermediate generation can pose an obstacle.

Due to these limitations, improvements to existing protocols seem crucial for further F_1_ breeding progress. Here, we provide theoretical and practical examination of the new protocol aimed at providing breeders with the ability to perform a higher number of line to line testing compared to existing protocols. The plant species chosen for practical evaluation of the protocol was white cabbage (*Brassica oleracea* L.), which is due to sporophytic incompatibility cross-pollinated species expressing inbreeding depression when made homozygous. However, the described protocol can be implemented also in hybrid breeding programs of self-pollinated species in which several approaches to prevent self-pollination.

The manuscript is composed of three parts; in the first part, the new breeding scheme is proposed; in the second part, theoretical calculations are provided for the number of F_1_ progenies needed to achieve a significant number of cross combinations; and in the third part, the whole procedure was tested in practical experiment with white cabbage using DH lines, their genotyping, maintenance, inter-pollination, field selection and paternity determination.

## Material and Methods

### Breeding Scheme

**Figure 1:**
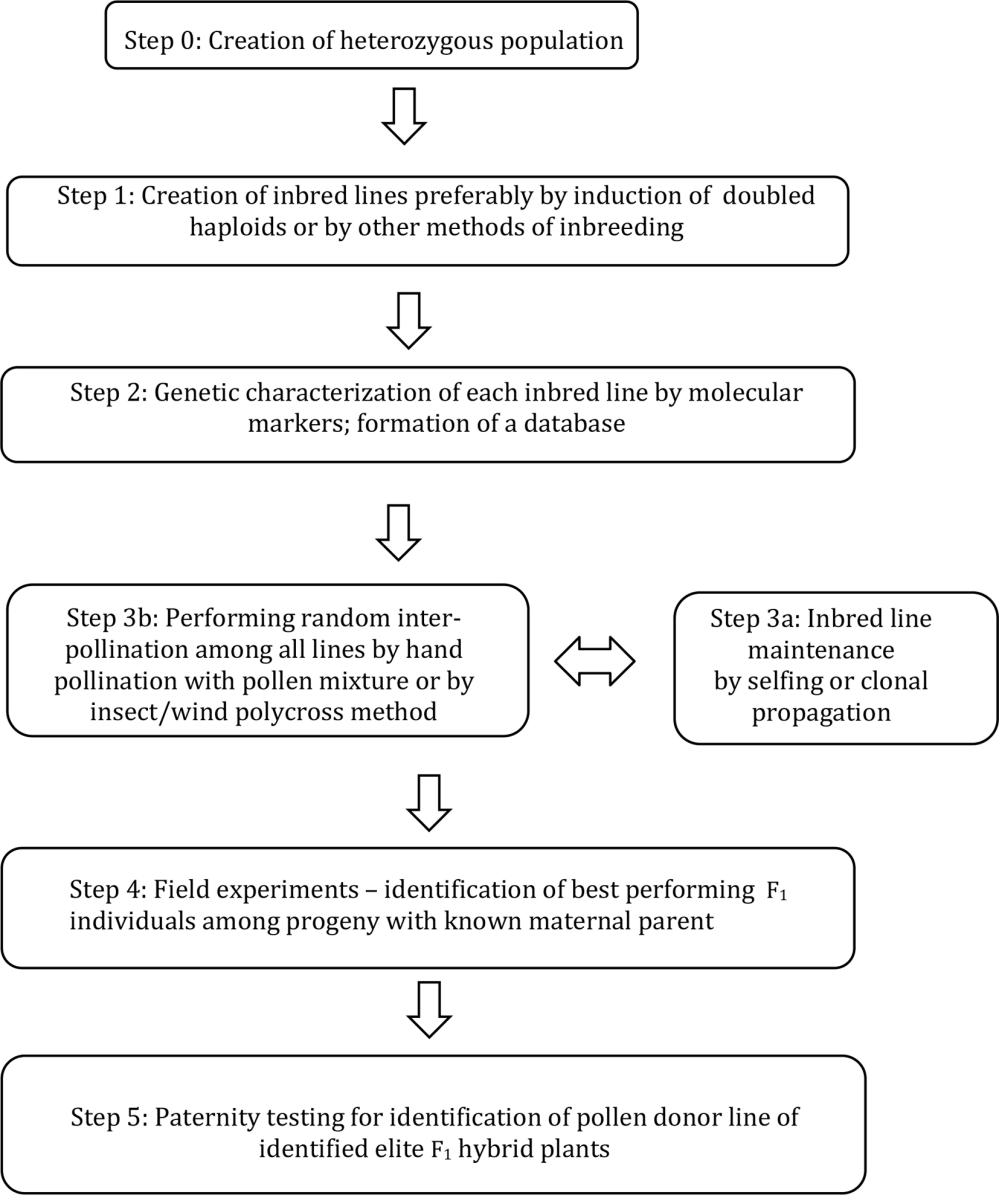
A method for combining ability testing of F_1_ hybrids by inter-pollination among genetically characterized DH lines and by revealing paternal origin of identified elite individuals within intercrossed progeny.

A new breeding method (**Figure 1**) was developed, supported by mathematical calculations and tested in a practical breeding protocol as described here.

Cabbage cultivars and DH were obtained from our cabbage hybrid breeding program, the list of genotypes included is given in **Supplementary Tables S1**, **S2**, **S3** and **S4**. In 2016, doubled haploid lines were induced from highly heterozygous donor plants (**Supplementary Tables S1** and **S2**) by microspore culture, as described earlier (Rudolf-Pilih et al., 2018). Genetically diverse DH cabbage seedlings were cultivated on a sterilised clay substrate (Klasmann-Deilmann) fertilised with Osmocote exact (15-9-12+2MgO+TE) in a greenhouse and exposed to low temperatures (0–5°C) for three months. During this period, each plant was genetically characterised by eight SSR markers as described in the next paragraph. Vernalised plants were induced to flowering in 2017. DH lines were maintained by selfing, which was efficient in only 6 of 69 lines due to self-incompatibility. For this reason, the majority of lines were maintained by micropropagation. Briefly, axillary buds were sterilised in 1.6% dichloroisocyanuric acid, washed in sterile doubled-distilled water and placed on MS medium containing 20 g l^-1^ sucrose, 8 g l^-1^ agar, 2 mg l^-1^ indolebutyric acid and 3 mg^-1^ benzylaminopurine. Shoots were subcultured on the same medium, while roots were induced on half strength MS medium lacking growth regulators.

**Figure 2:**
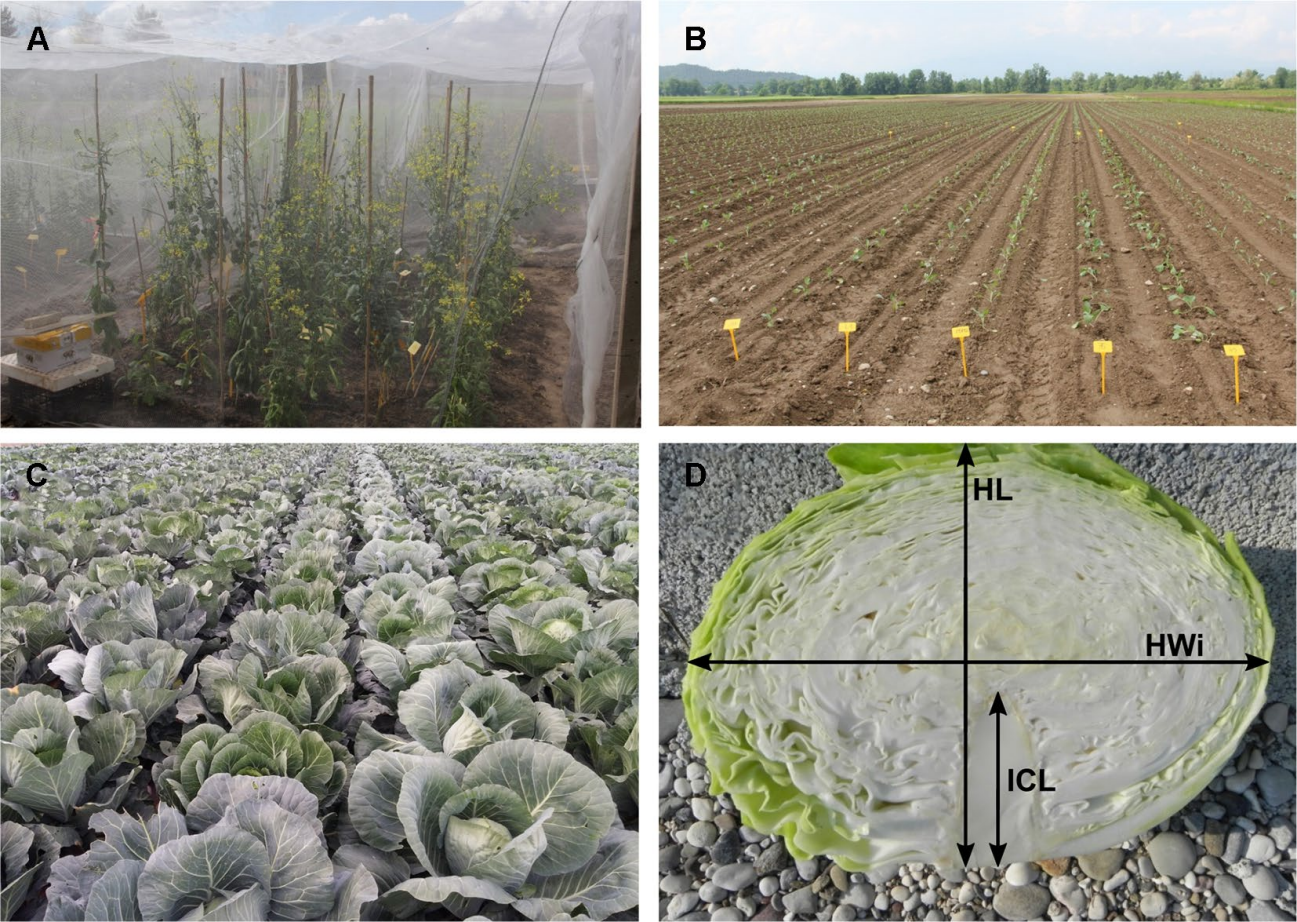
Illustration of selection process: **(A)** cage pollination using bumblebees **(B)** maternal-parent rows at transplanting **(C)** maternal parent rows at marketable maturity **(D)** head- cross section of representative hybrid: HL, head length; Hwi, Head width; ICL, inner core length.

For inter-pollination, two separate groups of DH lines were formed and inter-pollinated randomly by two different procedures. In the first procedure (cage pollination), 36 lines, each represented by a single plant, were placed into a cage at the beginning of flowering with bumblebees (*Bombus terrestris*) obtained from Koppert B.V. (Berkel en Rodenrijs, The Netherlands) (**Figure 2A**) until the end of flowering. In the second procedure (open pollination), 33 lines, each represented by a single plant, were placed on an isolated field and exposed to natural pollinators, predominantly bees. At maturity, pods were collected, dried and then seeds were scored and stored at 4°C until the next season. The selection of elite F_1_ hybrid plants was performed in 2018. Seeds were sowed at the end of March into a plug tray with 160 cells (60 trays in total) and seedlings were planted on the experimental field at the beginning of May and grown until marketable maturity. Fertilisation, irrigation and pest control were performed according to general agricultural practice. In total, 9,858 seedlings were grown with known maternal origin. All F_1_ hybrid descendants of a single maternal-parent plant were planted in a single row (**Figure 2B**). As previously described (Rudolf-Pilih et al., 2012) at maturity (**Figure 2C**), the following criteria were used to select elite F_1_ individual plants: head weight, head length, head width, length of inner core, head firmness and head shape (**Figure 2D**), while other characteristics like field resistance to *Xanthomonas campestris*, maturity, leaf colour and wax coating were also recorded. A leaf sample from each selected elite F_1_ plant was collected for DNA isolation and determination of paternity by SSR markers. DNA was isolated following the CTAB method (Doyle and Doyle 1987).

### Target SSRs and Design of Multiplex Primers for Genotyping

Eight microsatellite loci were included in the paternity test (**Table 1**), based on the power of distinction evaluated in the genotyping study of 352 cabbage genotypes (data available upon request). Amplification of eight microsatellites Kholilatul et al. (2014) (**Table 1**) was performed in a total volume of 15 μl containing 15 ng of DNA template, 1x PCR reaction buffer, 3.0 mM MgCl_2_, 0.8 mM of each dNTP, 0.45 unit *Taq* DNA polymerase; 0.15 µM of each primer (forward tailed primer and reverse primer). Each forward SSR primer has an universal M13 18 bp tail sequence added at 5’ end (5’-TGT AAA ACG ACG GCC AGT-3’) (Schuelke, 2000). Four different fluorescent dyes (6-FAM, VIC, NED, and PET) were used to label the four universal M13 primers which were included in single locus PCR at a concentration of 0.2 µM.

**Table 1:** SSR markers and characteristics of primers used in genetic analysis of white cabbage (Kholilatul et al., 2014).

Locus/Marker	Sequences 5’-3’	Motif
1. BoESSR825-for	GGACAGCGACACATTGAGTG	
BoESSR825-rev	GGGAAGAGGTTCCCAAACAT	(CCG)7
2. BoESSR391-for	GCGACCTGTTGAAGAAGGAG	
BoESSR391-rev	TTCTCCGCAAGAAATACAAGG	(GAT)7
3. BoESSR632-for	CCCTGCAATTGAAAACCAGT	
BoESSR632-rev	AAACCGTCCAAGGATCATCA	(TGT)7
4. BoESSR492-for	GCGCAGAATCCAGATCATAG	
BoESSR492-rev	GGCTGGAGTATGAGCGAGAC	(GA)9
5. BoESSR338-for	TGTAGCCGAAAGGGAATGAG	
BoESSR338-rev	GTGCTTGCATCCAGAAACCT	(AC)10
6. BoESSR484-for	ACCCATACGTCCACGTCAAT	
BoESSR484-rev	GCAATCGTCTTTCCACCAAT	(AGA)7
7. BoESSR087-for	GTTTCCTCTTCCACCACCAA	
BoESSR087-rev	AATCTATCAAGAGGGCCAAGG	(TCC)7
8. BoESSR053-for	TTTGCCAAGAAGCCTGAAGT	
BoESSR053-rev	TGTACCAGCTGCAACCTCTG	(GAA)7

The cycling conditions were as follows: 95°C for 5 min; 10 cycles of 30 s at 95°C, 30 s at 65°C, which was lowered by 1°C in each cycle, and 30 s at 72°C; 25 cycles of 30 s at 95°C, 30 s at 55°C, and 30 s at 72°C; and a 5-min extension step at 72°C. Samples were kept at 4°C until analysis.

The PCR products amplified with 4 different dyes were mixed together and same amount of formamide was added with GeneScan 600 LIZ size standard, heat denatured, chilled on ice and run on a capillary electrophoresis system ABI 3730XL analyser (Applied Biosystems). Resulting electropherograms were analysed using GeneMapper 4.0 (Applied Biosystems) or PeakScanner software (Applied Biosystems), where the length of alleles was recorded.

### Paternity Assignment

Using likelihood ratios, determination of paternity by CERVUS 3.0.7 software was calculated (Kalinowski et al., 2007). Based on genotyping data with eight microsatellites, the following parameters of genetic variability and information content were calculated for all 282 genotypes analysed.

Parentage analysis was performed in two steps, in the simulation of parentage analysis and actual parentage analysis. Simulation was run to estimate the resolving power of a series of SSR loci; simulation parameters were as follows: 10,000 progenies, the number of candidate parents set to 36 for cage pollination and 33 for open pollination, the proportion of sampled parents assumed to be one, proportion of loci typed 0.998 and proportion of loci mistyped 0.002. Actual parentage analysis was performed to test candidate parents against offspring and, for each offspring tested, to assign the most-likely candidate parent with a pre-determined level of confidence. Finally, paternity analysis was done as maternal genotypes were available and 100% confirmed with parentage analysis. The objective was to assign a male parent to each offspring. The overall likelihood ratios were expressed as logarithm of odd (LOD) scores (natural logarithm of the overall likelihood ratio) and were assigned to each possible parent and parent pair. When the trio LOD score had a probability >95% (strict) or >80% (relaxed) based on confidence derived from prior parentage simulation, and there were no parental marker discrepancies, the candidate parents were assigned to the progeny plant. CERVUS only assigned paternity if at least eight out of eight loci were scored.

### Mathematical Calculations

For calculation of line/offspring probabilities, Wolfram Mathematica v. 11 was used, as discussed in Results point B.

## Results

### A. Breeding Scheme “Inter-Pollination/Progeny Testing”

A breeding scheme, aimed to increase the efficiency of inbred testing for hybrid development is proposed. Briefly, from a heterozygous starting population, DH lines are produced and genotyped. A selected group of diverse lines (determined by molecular markers) is inter-pollinated and their F_1_ progeny evaluated in field experiments. It is expected that, by chance, in the progeny of random crosses between inbred lines individual elite plants are encountered, carrying a highly desirable set of allelic combinations. These individual F_1_ hybrid plants, being heterozygous and unique, are identified by their phenotypes. Since each inbred line is genetically characterised both parents are identified and confirmed by molecular marker analysis.

The scheme of the method for breeding hybrid plants is given in **Figure 1**; a more detailed explanation of each step is given below:

Step 0: Establishment of heterozygous starting population due to the breeding goals.

Step 1: Producing essentially homozygous donor lines from starting population. This can be optimally achieved by induction of doubled haploids, but other methods of inbreeding can also be considered.

Step 2: Each DH line is genetically characterised by means of molecular markers to obtain a unique genetic profile for each line. For each group of lines entering step 3, a unique database is formed.

Step 3a: Each DH line is maintained either by selfing (if achievable) or clonally propagated by various means including micropropagation.

Step 3b: Inbred lines are induced to flower simultaneously and random inter-pollination is stimulated by various methods to obtain F_1_ hybrid progeny.

Step 4: F_1_ hybrid progeny is sown and the maternal origin for each seed is recorded (»maternal-parent rows«). Elite F_1_ hybrid individuals are identified by phenotyping among the F_1_ progeny;

Step 5: Paternity of selected elite individual plants is determined by molecular markers using specific software by comparison to the DH line database formed in Step 2.

The number of lines entering inter-pollination is limited by practical reasons such as pollination characteristics of plants and the ability of breeders to maintain lines and perform analysis. For most cases, we propose that the number of lines within one group will not exceed 100. In the case that more lines need to be tested, a two-step procedure is proposed. In the first step, a group of, say, 100 lines are tested according to the scheme in **Figure 1**, and at the same time additional groups are tested in the same way. Identification of a particular DH that produced numerous selected hybrid plants in cycle 1, would be an indication of positive general combining ability. These good combining DHs can be intercrossed in a second cycle to identify superior hybrids.

### B. Theoretical Calculation of the Size of F1 Families Needed to Obtain a Given Probability of Cross Combinations Following Inter-Pollination Among Inbred Lines

Inter-pollination among selected inbred lines by previously explained methods produces F_1_ progeny that should in the optimal case represent all possible combinations. The probability of achieving all crossing combinations is related to the number of lines entering inter-pollination and the number of seeds obtained and sown by each inbred line. We developed a mathematical model that can be used for the calculation of these probabilities. We also examined consequences of the number of F_1_ seeds needed in case the breeder is satisfied with some missing combinations - for instance with 90 or 80% one-way crossing representation per maternal-parent row.

**Assumptions**: A set *L* = {*v*_1_, *v*_2_,…, *v* *_n_*} of *n* distinct inbred lines of a plant species is given. For each *i* {1,2,…,*n*}, *k* plants of line *v* *_i_* are grown and successfully pollinated by a mixture containing pollen from each of the lines v_1_, v_2_, ..., v_n_ . It is assumed that the probability of successful pollination of v_i_ by v_j_ is independent of i and j.

**Question:** We wondered what the probability is for *n* lines in the F_1_ offspring that, in an individual row with the same maternal-parents, at least *m* of the paternal-parents are present at least once. More precisely, what is the probability Q(*n*, *k*, *m*) that a fixed maternal-parent line *v* *_i_* will be pollinated by at least *m* paternal-parent lines different from *v* *_i_*?

To answer this, we model our experiment by the process of selecting, uniformly at random, an element from the set *L* *^k^* of all strings of length *k* over the alphabet *L*. First, we fix a subset *L* *_j_* ⊆ *L* \ {*v* *_i_*} of *j* paternal-parent lines, and enumerate the set *M* *_j_* of all those strings of length *k* over *L* *_j_* ∪ {*v* *_i_*} in which each of the lines from *L* *_j_* appears at least once. Clearly *M* *_j_* = *M’* ∪ *M’’* where *M’* is the set of those strings from *M* *_j_* which do not contain *v* *_i_*, and *M’’* the set of those strings from *M* *_j_* which do contain *v* *_i_*. Then, *M’* is in a one-to-one correspondence with the set of all surjective maps from the set {1, 2,…, *k*} onto *L* *_j_*, and *M’’* is in a one-to-one correspondence with the set of all surjective maps from {1, 2,…, *k*} onto *L* *_j_* ∪ {*v* *_i_*}, so:

$$\text{|}M_{j}\text{| }=\text{ |}M'\text{| }+\text{ |}M''\text{| }=j!S_{k,j}+\text{ (}j+\;1\text{)}!S_{k,j+1}$$

(1) $$=j!\text{ (}S_{k,j}+\text{ (}j+1\text{)}S_{k,j+1}\text{) }=j!S_{k+1,j+1}$$

where *S* *_k,j_* denotes the Stirling number of the second kind, enumerating the set of all partitions of a *k-*element set into *j* non-empty blocks. Since a set *L* *_j_* ⊆ *L* \ {*v* *_i_*} can be chosen in $\left(\begin{array}{c} n-1\\ j \end{array}\right)$ ways, formula (1) implies that the cardinality of the set *M* *_j,k,n_* of all those strings *L* *^k^* in which *exactly j* lines different from *v* *_i_* appear is:

$$\text{|}M_{j,k,n}\text{| }=\;\left(\begin{array}{c} n-1\\ j \end{array}\right)j!S_{k+1,j+1}$$

Therefore, the number of strings from *L* *^k^* in which at least *m* lines different from *v* *_i_* appear is equal to:

$$\sum_{j=m}^{n-1}\left(\begin{array}{c} n-1\\ j \end{array}\right)j!S_{k+1,j+1}$$

and the probability that at least *m* paternal-parent lines distinct from *v* *_i_* will be represented in the offspring is:

(2) $$Q(n,k,m)=\frac{1}{n^{k}}\sum_{j=m}^{n-1}\left(\begin{array}{c} n-1\\ j \end{array}\right)j!S_{k+1,j+1}$$

which is the answer to the question posed.

**Tables 2** and **3** exhibit some values (rounded to three decimal places) obtained from formula (2) for the probability that given a fixed maternal-parent line, *v* *_i_*_,_ at least *m* paternal-parent lines will appear in the F_1_ seeds.

**Table 2:** Probabilities *Q*(*n*, *k*, *m*) for *n* = 61 and *m* = 60, 54, 48.

k	Q(61, k, 60)	Q(31, k, 54)	Q(31, k, 48)
60	0.000	0.000	0.000
120	0.000	0.216	0.969
180	0.032	0.979	1.000
240	0.304	1.000	1.000
300	0.650	1.000	1.000
360	0.854	1.000	1.000
420	0.943	1.000	1.000
480	0.979	1.000	1.000
540	0.992	1.000	1.000
600	0.997	1.000	1.000
660	0.999	1.000	1.000
720	1.000	1.000	1.000

**Table 3:** Probabilities *Q*(*n*, *k*, *m*) for *n* = 31 and *m* = 30, 27, 24.

k	Q(31, k, 30)	Q(31, k, 27)	Q(31, k, 24)
30	0.000	0.000	0.003
60	0.004	0.340	0.925
90	0.173	0.947	1.000
120	0.540	0.999	1.000
150	0.799	1.000	1.000
180	0.921	1.000	1.000
210	0.970	1.000	1.000
240	0.989	1.000	1.000
270	0.996	1.000	1.000
300	0.998	1.000	1.000
330	0.999	1.000	1.000
360	1.000	1.000	1.000

### C. Elaboration of »Interpollination/Paternity Testing« Method in White Cabbage

#### Interpollination Among DH Lines

Interpollination was successful in both pollination methods. It should be noted that since each DH was represented just as a single plant, suboptimal representation of each genotype can be expected. We found that from 36 and 33 different DHs in cage or open field selections, respectively, 23 and 18 paternal parents were represented in their elite F_1_ progeny. It is most likely that progenies of some paternal DHs were not chosen as elite because of undesired phenotypic characteristics.

#### Selection Procedure

In total, 5,018, and 4,840 F_1_ hybrid plants resulting from cage pollination or open pollination, respectively, were planted, of which 126 and 87 were selected based on their phenotypic characteristics (**Table 4** and **Table 5**). In general, major differences in phenotypic characteristics were found but, as expected for half-sib families, also some similarities among plants within maternal-parent rows (**Figure 2**).

**Table 4:** List of maternal-parents and the determined paternal-parents within F1 progeny, logarithm of odd (LOD) score and number of selected plants with the same parent line for cage pollination experiment.

DH plant line line No. (maternal parent)	Determined paternal parent	LOD	No. of selection with the same	
1	11	5.80	1	*rec
	28/281	5.17	1	
11	11	7.58	1	
	79	6.27	2	
	192	8.67	1	*rec
28	1	7.58	2	
	59	5.51	1	
	79	6.27	1	
	272	7.71	3	
	311	9.46	1	*rec
40	275	9.51	3	
43	11	5.79	1	
	341	5.85	6	*rec
48	1	7.57	1	
	272	7.71	1	
	311	9.45	1	
52	43	6.79	1	
	261	7.19	2	
	342	6.16	1	
65	52	6.63	1	
	121	7.90	4	
121	261	7.19	1	
	311	9.45	2	*rec
	341	5.85	1	
189	79	6.27	1	
	275	9.51	1	
192	11	5.80	3	
	79	6.27	1	
	311	9.46	2	*rec
210	11	5.79	3	
	311	9.45	1	*rec
236	261	7.19	1	
	311	9.45	1	
249	79	6.27	1	
	121	7.90	2	
	341	5.85	2	*rec
261	79	6.27	1	
	341	5.85	19	*rec
272	1	7.58	1	
	121	7.90	1	
	236	8.66	1	
274	121	7.90	1	
275	121	7.90	1	
276	249	6.39	1	
281	1	7.58	1	
	11	5.80	5	
	79	6.27	1	
	275	9.51	1	
	311	9.46	2	*rec
311	11	5.80	2	
	28/281	5.17	2	
	104	7.89	1	
	121	7.90	4	
	192	8.68	1	
	210	5.58	1	
	249	6.39	2	
	346	8.50	1	
341	43	6.79	3	
	52	6.63	1	
	65	7.62	1	
	79	6.27	1	
	249	6.39	1	
	261	7.19	9	
342	11	5.79	1	

*reciprocal.

**Table 5:** List of maternal-parents and the determined paternal-parents within F1 progeny, logarithm of odd (LOD) score and number of selected plants with the same parent line for open pollination experiment.

DH plant line line No.(maternal parent)	Determined paternal parent	LOD	No. of selection with the same parental lines	
7	164	6.67	1	
	260	6.92	1	
	349	11.30	1	
15	49	7.71	1	
	176	5.88	2	
	247	6.29	1	
75	171	6.82	2	
	306	8.01	1	
	349	11.30	1	
85	9	7.95	2	
	49	7.71	5	
103	176	5.88	1	
	260	6.92	1	
123	49	7.71	2	
	176	5.88	1	
	273	9.76	2	
	349	11.30	4	
164	9	7.95	1	
168	26	9.47	1	
	247	6.29	1	*recip
	292	6.70	1	
176	35	7.66	1	
	247	6.29	1	*recip
247	9	7.95	1	
	168	8.15	2	
	176	5.88	2	
	253	8.31	1	
	273	9.76	1	
253	260	6.92	1	
	304	8.91	1	
	306	8.01	3	*recip
	344	6.90	1	
260	273	9.75	1	*recip
	292	6.70	3	*recip
273	253	8.31	3	
	260	6.92	1	
	292	6.70	1	
292	49	7.71	1	
	164	6.67	1	
	260	6.91	6	
	306	8.01	2	
	349	11.30	1	*recip
304	13	7.14	1	
	247	6.29	2	
	264	7.68	3	
306	164	6.67	2	
	253	8.31	2	
	292	6.70	1	
344	306	8.01	3	
347	9	7.95	2	
	49	7.71	1	
349	49	7.71	2	

*reciprocal.

In both pollination groups, a relatively large number of plants selected in the field according to their phenotypic characteristics had identical parents (**Table 4** and **Table 5**). Also, several reciprocal genotypes were found (48 in total), meaning that plants with the same genotype but spread across the selection field were recognised as elite within rows with different maternal-parents. This finding confirms, that based on phenotypic selection of individual plants at least in white cabbage selection is efficient.

Morphological characteristics of six chosen superior hybrids and the standard variety ‘Presnik F1’ are given in **Table 6**.

**Table 6:** Selected potential super elite F_1_ plants based on head morphological characteristics, compared to the released cultivar ‘Presnik F_1_’.

Mother plant	Male parent	Average head weight (kg)	Average length of inner core (cm)	Average head shape*	Average head firmness**	Figure
261	341	3.78	9.3	0.7	5	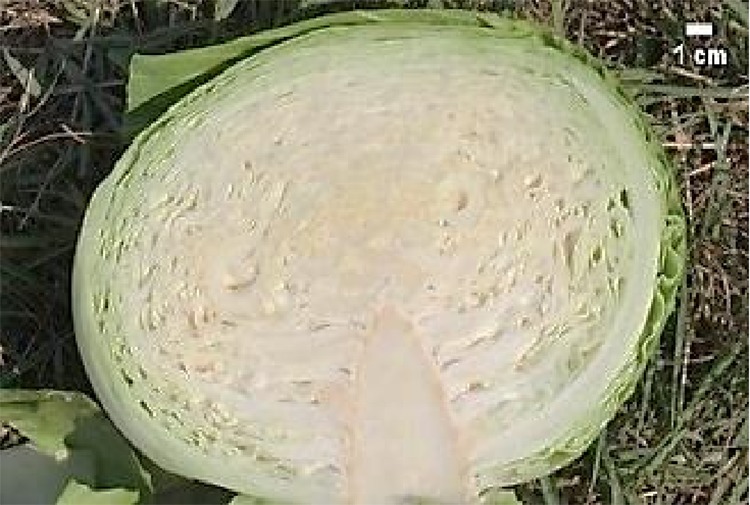
192	11	3.97	8.0	0.6	5	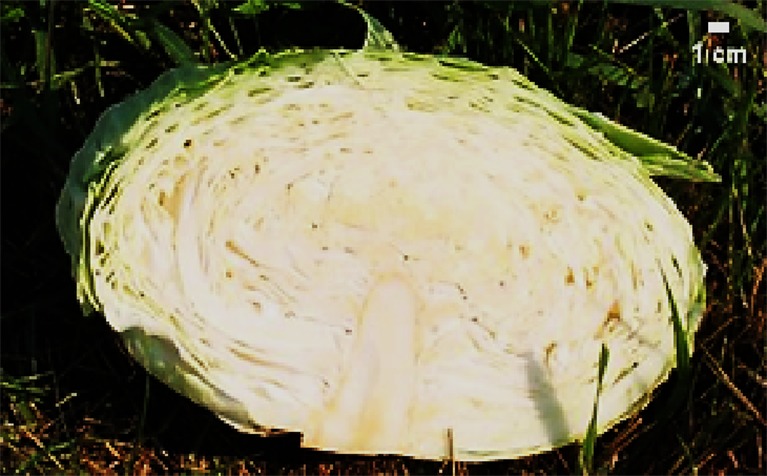
341	52	3.13	6.5	1.0	5	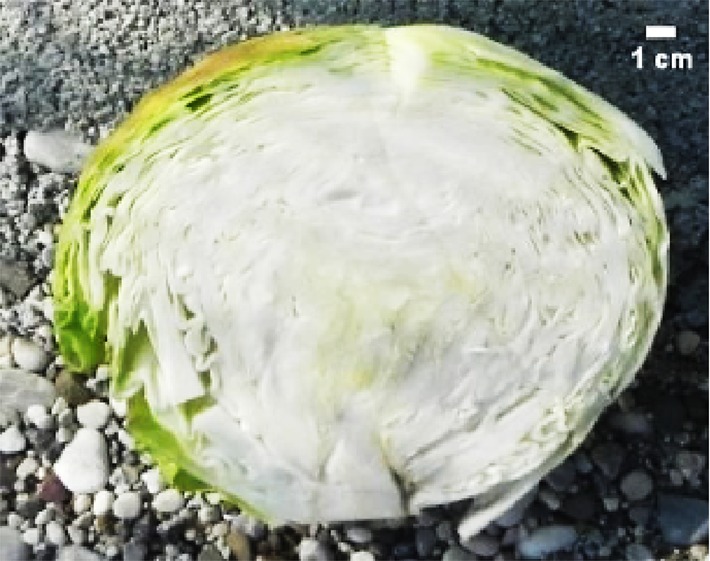
253	304	3.35	8.0	0.7	5	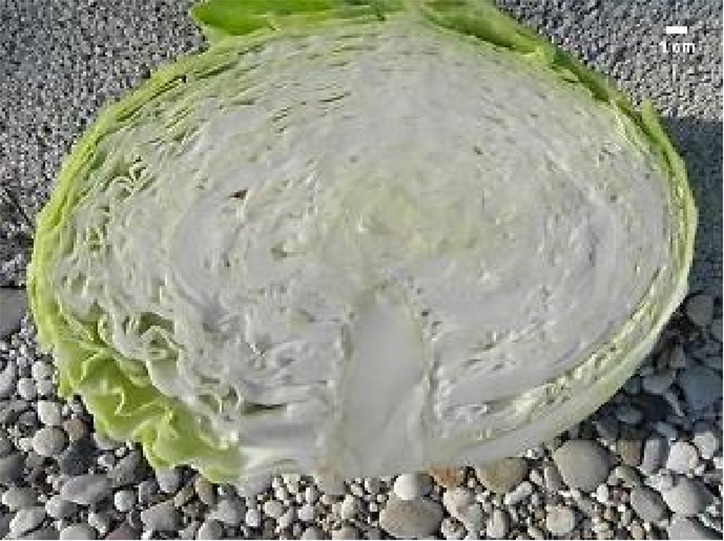
85	49	3.67	7.5	0.7	5	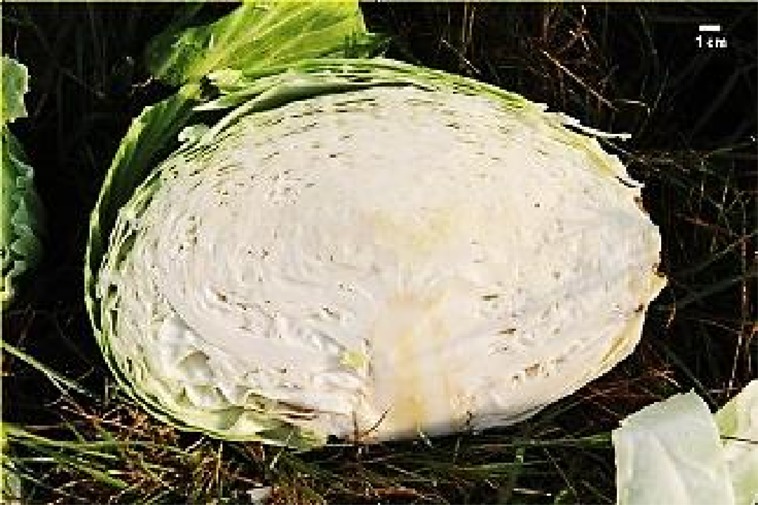
Hybrid Presnik F1		2.60	7.5	0.9	5	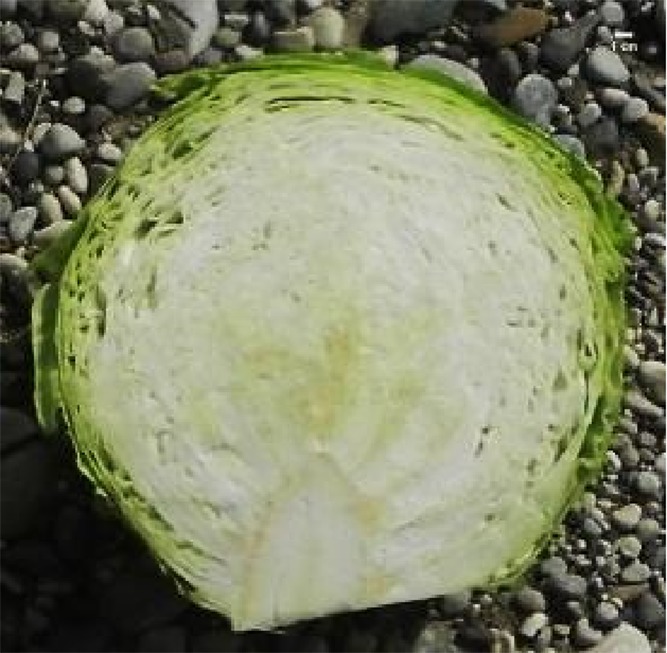

*quotient between height and width number lower than 1 - flattened, over 1 - round.

**firmness: 1 (low) 5 (highest).

### Determination of Paternity

Eight SSR loci used for genotyping produced distinct patterns among 36 and 33 inbred lines of both cage and open pollinated groups, respectively. The only exceptions were two lines (line 28 and line 281) which exhibited the same allelic structure (**Supplementary Tables S5** and **S6**).

Variability parameters were calculated for eight loci on the whole set of data; microsatellites were successfully amplified in all 282 genotypes, and a total of 29 different alleles were detected. The average number of alleles per locus was 3.625, and, the number of amplified alleles at each locus varied from 2 (locus 6) to 5 (loci 2, 4, 5). The highest observed heterozygosity (0.631) was found at locus 5 and the lowest at locus 6 (0.089) (**Supplementary Table S7**). In spite of low heterozygosity, locus 6 has additional value in distinguishing all of the parent lines in combination with the other seven loci.

Parentage analysis based on 8 microsatellite markers resulted in a panel of parent/offspring trios LOD (**Table 4**, **Table 5**). The LOD score value was used for determination of the threshold that validates the hypothesis and for discarding trios with high allelic discrepancies.

In the case of cage pollination, the strict LOD threshold (95% confidence) was at LOD >5.35 and the correct paternity assignment was achieved in all of them. In the case of open pollination, the LOD threshold was at LOD >5.28 and the correct paternity assignment was also achieved in all of them.

Among the 213 elite F_1_ plants tested, 99 were found to be genetically diverse, while others were identical, including those formed by reverse combination. Out of these, 5 hybrids were selected as super elite. These super elite F1 hybrids are now entering multiplication and additional field testing to be registered as new varieties.

## Discussion

The main characteristic of the method presented is built around paternity determination of the best selected individual F_1_ plants originating from the inter-pollination of inbred lines. Using molecular markers, individual plants with distinguished genotypes can be clearly identified. In our case, eight SSR markers were sufficient to discriminate between all but one included cabbage inbred lines. Since maternal-parent origin of lines was recorded, only paternal-parent lines of elite hybrids needed to be identified.

Paternity analysis is used extensively in molecular evolution, molecular ecology and forensic science. For this purpose, software applications were developed such as CERVUS. The aim of this test is to identify paternal identity. This approach was used to identify paternal origin of Chinese Holstein cattle (Tian et al., 2008). Nine selected polymorphic SSR markers efficiently discriminated the parental origin of 330 genotypes.

The idea of using paternity testing in plant breeding schemes was first presented (Lambeth et al., 2001) in loblolly pine (*Pinus taeda* L.). This method was later applied in several other tree species, for instance in *Eucalyptus* sp. (Cupertino et al., 2009) and in olives (Baruca-Arbeiter et al., 2014). Paternity testing was also used to identify pollen contamination rate, for instance in loblolly pine (Vidal et al., 2015). Under the term “breeding without breeding” paternity determination was proposed for forest tree breeding protocols (Wang et al., 2010). It is more difficult to identify paternal origin in tetraploid allogamous plants; nevertheless, such an approach was achieved in some forage plant species, particularly with the help of software applications developed specifically for these needs. Such applications were developed (Spielmann et al., 2015) and used in autotetraploid species such as alfalfa. Paternity testing was also proposed in alfalfa breeding programs (Riday, 2011; Riday et al., 2013). It should be noted that in alfalfa polycross breeding, producing synthetic cultivars but no F_1_ hybrids is practiced. For these reasons, alfalfa and other forage crop breeders do not select individual homozygous lines or genotypes in search for F_1_ hybrid performance, as discussed in this manuscript.

Despite the evident success of F_1_ hybrid varieties in crop plants, vegetables and ornamentals, the major features of breeding methods have not been changed for several decades. So far, alternative attempts like the already described “reverse breeding” (Dirks et al., 2009) have not gain major attention. In these standard methods, testing for combining ability is often the limiting factor. As stressed by Acquaah (2012), even testing for specific combining ability among 50 lines requires 2,450 crosses, which is too laborious and technically demanding to be performed. Experimental data support this assumption. For instance in a large trial with *Artemisia annua* (Townsend et al., 2013) attempting to perform diallele cross among 30 lines, the authors succeeded in obtaining only 366 that yielded enough seed to be grown up for screening (156 of these were reciprocals) from 870 possible reciprocal cross combinations. Several other authors report on even much lower numbers of SCA testing.

In our “inter-pollination/paternity testing” method, inter-pollination among all inbred lines is an option, since the origin of offspring is later determined by genetic profiling. The method is much less laborious since genetic profiling is done only on inbred lines entering inter-pollination and only on previously phenotypically selected elite F_1_ hybrids. This was also shown in our experiment with cabbage, where 69 parental lines and only 213 F_1_ hybrids from 9,858 evaluated needed to be genotyped in total.

In cabbage, accidental self-pollination is not frequent due to strong sporophytic self-incompatibility. For instance, in our test, we found only three inbreds within 104 detected experimental hybrids (data not shown). It is a key characteristic of inbred lines to be less vigorous than hybrids. For this reason it was also shown in our experiments where no accidentally self-pollinated DH line was selected among elite F_1_ hybrids.

In the case that the proposed breeding strategy will be implemented for self-pollinating species (hybrids already dominate market in rice and became important in wheat and barley), the optimal pollination method used will differ from species to species. Various solutions can be proposed. To perform interpollination of self-pollinating species we propose pollen collection from each inbred line followed by and manual pollination of emasculinated flowers with pollen mixture.

It might be discussed whether selection based on individual F_1_ plant performance is adequate. Major traits with high heritabilities, such as those used in our trial with cabbage, are optimal for selection. Although the overall performance of selected individual F_1_ hybrid combinations will be adequately tested in the next growing seasons, two indications show that phenotypic selection among individuals is likely to be efficient. Namely, paternity analysis showed, that in both pollination methods several F_1_ hybrids with the same parental inbred lines were selected (**Tables 4** and **5**). Also, in 48 cases, the same F_1_ combination was selected, but in a reciprocal way, meaning that the selection of these F_1_ most likely based on phenotype was adequate. These observations would be an indication of good general combining ability, which can be tested by intercrossing among DHs in subsequent studies.

Advances in phenotypic analysis achieved during recent years using image analysis methods usually called plant phenomics (Afonnikov et al., 2016) might even improve the selection process. Using specific software and computer image analysis, individual plants are tested (among others) concerning development, water use, architecture, shapes and reflectance at a wide range of wavelengths, from visible light to heat imaging. This testing can be performed in both controlled and field conditions (White et al., 2012).

Using a formula that was developed to calculate how many F_1_ offspring have to be screened after such random crosses, it is now possible to predict the number of F_1_ offspring needed per maternal-parent row. It would be optimal if all of the lines were crossed among each other and their progeny evaluated. Even if we assume that pollination was equally successful and that all plants were inter-pollinated, the probability that each progeny is presented at least once in a maternal-row increases with the number of lines being inter-pollinated. For instance, according to **Table 2**, when 30 DH lines are inter-pollinated, the probability *p* = 0.996 that all possible combinations will occur among F_1_ progeny is achieved with 270 F_1_ plants per maternal-parent row. However, if the breeder accepts that only about 90 or 80% of possible combinations are expected in F_1_ progeny, the probability *p* = 0.999 or *p* = 1.000 that this criterion is satisfied is reached already with 120 or 90 F_1_ plants, respectively. Similar conclusions can be drawn in the case when 60 DH lines are intercrossed (**Table 3**). The probability *p* = 0.992 that all possible combinations will occur among F_1_ progeny is reached with 540 F_1_ plants per maternal-parent row. If only 90% or 80% of possible combinations are expected, the probability *p* = 1.000 is achieved with 240 or 180 F_1_ plants, respectively.

It should be noted that probabilities are given per maternal-parent line while reciprocal effects are not considered. If we neglect »maternal effects« caused by differences in cytoplasmic inheritance, the probability that the same combination of DH lines occurs within other rows because of reciprocity is significant. If this is taken into account for practical considerations, the number of F_1_ plants examined might be even lower.

It is a characteristic of white cabbage that all experimental conditions needed for evaluation of the presented breeding method are well established, namely the procedure of DH induction yields large numbers of haploid embryos that frequently spontaneously double (Hong-Hao et al., 2014), DH lines are routinely selfed or maintained by micropropagation, the SSR marker analysis is well established (Wang et al., 2012) resulting in a high level of polymorphism, and phenotypic selection of most valuable characteristics can be performed. In our experiments, we found no major obstacle and all lines constituting selected F_1_ hybrids were maintained.

A more complex polycross pollination method, which usually contains 10 or more repetitions of each genotype, would certainly increase the representation of genotypes, but would require an additional year to micropropagate each DH line. The benefits of both options need to be further evaluated.

## Conclusion

Here we propose a novel F1 hybrid breeding method which is composed of already known elements such as DH extraction, genotyping, intercrossing, line maintenance and paternity testing. New is the combination of these elements that provide higher efficiency and is less labour intensive.

Selection based on individual plants can result in the overestimation of some selected F_1_ plants because of their favourable position. Although the selection of elite plants by chance cannot be excluded and could be tested only by repeated pollination and testing of selected parental lines, two indications suggest otherwise. The fact that we collected several elite hybrid plants with identical parents and the fact that in addition to this several of them were found across the selection field as being reciprocals indicate that selection based on individual plants by phenotype is most likely efficient. Besides reducing the time and labour needed for crossing, the proposed breeding scheme favours the selection of parent lines which flower simultaneously, are compatible and produce higher amounts of seeds, all of which is very important in breeding programs.

We believe that the proposed scheme could be easily adopted for other vegetable or crop species. Of course all aspects of the proposed technique need to be widely tested and verified both for practical and economic criteria.

## Data Availability Statement

All datasets generated for this study are included in the manuscript/**Supplementary Files**.

## Author Contributions

JJ, KR-P and BB designed the research; MP, KR-P, BB, NŠ and JM performed the research and analysed the data; BB wrote the manuscript with input from MP, KR-P, NŠ and JM.

## Funding

The authors acknowledge the financial support from the Slovenian Research Agency (research core funding No. P4-0077) and the funding of the project by the Ministry of Agriculture, Forestry and Food (No. 08-6-72/2017).

## Conflict of Interest

The authors declare that the research was conducted in the absence of any commercial or financial relationships that could be construed as a potential conflict of interest.
