# Supplementary material for: Proposal of a New Hybrid Breeding Method Based on Genotyping, Inter-Pollination, Phenotyping and Paternity Testing of Selected Elite F1 Hybrids
Source: Front Plant Sci. 2019 Sep 18;10:1111. doi: 10.3389/fpls.2019.01111 (PMC6759491; doi:10.3389/fpls.2019.01111)
Supplement: Supplementary file 1 [file DataSheet_1.pdf]

Table S1: The origin of inbred lines used in F1 hybrid production in cage experiment and genotyping with eight molecular markers

genotypes	donor plant	locus 1		locus 2		locus 3		locus 4		locus 5		locus 6		locus 7		locus 8	
1	Burton F1	241	241	371	371	135	135	202	202	153	153	275	275	162	162	273	273
11	Autumn queen F1	241	241	325	325	150	150	196	196	145	145	275	275	162	162	273	273
28	92*Atria F1	241	241	374	374	150	150	196	196	153	153	275	275	162	162	253	253
40	Krautman F1	237	237	325	325	135	135	208	208	153	153	275	275	155	155	273	273
43	4*Atria F1	237	237	325	325	150	150	208	208	153	153	275	275	155	155	253	253
48	Atria F1*6	241	241	325	325	150	150	206	206	153	153	275	275	155	155	253	253
52	Autumn queen F1	241	241	374	374	150	150	196	196	145	145	275	275	162	162	273	273
53	Kranjsko okroglo*(36*165)	237	237	325	325	150	150	206	206	148	148	275	275	155	155	253	253
59	Atria F1*6	241	241	325	325	150	150	202	202	148	148	275	275	155	155	253	253
65	278*Burton F1	237	237	371	371	150	150	202	202	153	153	275	275	162	162	273	273
76	Kranjsko okroglo*(36*165)	241	241	368	368	150	150	206	206	148	148	275	275	155	155	253	253
79	Krautman F1	241	241	325	325	135	135	202	202	148	148	275	275	155	155	253	253
99	Kranjsko okroglo*5	241	241	374	374	135	135	206	206	153	153	275	275	155	155	273	273
104	92*Atria F1	241	241	348	348	150	150	202	202	153	153	275	275	155	155	273	273
105	Kranjsko okroglo*5	241	241	348	348	135	135	206	206	153	153	275	275	155	155	253	253
121	5*Atria F1	241	241	325	325	150	150	202	202	143	143	275	275	150	150	253	253
181	92*Kranjsko okroglo	241	241	374	374	150	150	208	208	153	153	275	275	162	162	273	273
189	Kranjsko okroglo*5	241	241	374	374	150	150	206	206	145	145	275	275	155	155	253	253
192	Kranjsko okroglo*5	241	241	348	348	150	150	206	206	153	153	275	275	155	155	253	253
198	Kranjsko okroglo*5	241	241	348	348	135	135	202	202	153	153	275	275	155	155	273	273
210	Kranjsko okroglo*7	237	237	325	325	150	150	196	196	153	153	275	275	155	155	253	253
236	Atria F1*5	241	241	325	325	135	135	202	202	143	143	275	275	150	150	253	253
240	7*Atria F1	237	237	325	325	150	150	208	208	153	153	275	275	155	155	273	273
243	8*Atria F1	237	237	368	368	150	150	196	196	153	153	275	275	162	162	253	253
249	2*Atria F1	241	241	374	374	150	150	208	208	153	153	275	275	162	162	253	253
261	7*Atria F1	237	237	325	325	150	150	196	196	148	148	275	275	155	155	273	273
265	8*Atria F1	237	237	368	368	150	150	196	196	148	148	275	275	162	162	253	253
272	278*Burton F1	237	237	371	371	135	135	196	196	153	153	275	275	162	162	253	253
274	278*Burton F1	241	241	371	371	150	150	196	196	153	153	275	275	162	162	273	273
275	278*Burton F1	241	241	325	325	135	135	202	202	50	50	285	285	162	162	253	253
276	92*Atria F1	241	241	374	374	135	135	202	202	153	153	275	275	155	155	253	253
281	8* Atria F1	241	241	374	374	150	150	196	196	153	153	275	275	162	162	253	253
311	Grandslam F1	-	-	374	374	150	150	210	210	145	145	275	275	162	162	253	253
341	278*Burton F1	241	241	325	325	135	135	202	202	50	50	275	275	162	162	253	253
342	Kranjsko okroglo*7	237	237	325	325	150	150	202	202	145	145	275	275	155	155	253	253
346	Kranjsko okroglo*165	237	237	368	368	150	150	202	202	153	153	275	275	155	155	253	253
