# Supplementary material for: Proposal of a New Hybrid Breeding Method Based on Genotyping, Inter-Pollination, Phenotyping and Paternity Testing of Selected Elite F1 Hybrids
Source: Front Plant Sci. 2019 Sep 18;10:1111. doi: 10.3389/fpls.2019.01111 (PMC6759491; doi:10.3389/fpls.2019.01111)
Supplement: Supplementary file 2 [file DataSheet_2.pdf]

Table S2: The origin of inbred lines used in F1 hybrid production in open pollination experiment and genotyping with eight molecular marker

genotype	donor plant	locus 1		locus 2		locus 3		locus 4		locus 5		locus 6		locus 7		locus 8	
7	Fieldwinner F1	241	241	325	325	150	150	210	210	153	153	275	275	155	155	253	253
9	Benelli F1	70	70	325	325	150	150	210	210	145	145	275	275	155	155	273	273
13	Kranjsko okroglo*5	241	241	374	374	135	135	202	202	145	145	275	275	155	155	253	253
15	Kranjsko okroglo*92	237	237	374	374	150	150	208	208	153	153	275	275	155	155	253	253
16	Kranjsko okroglo*92	241	241	374	374	150	150	208	208	145	145	275	275	162	162	253	253
26	278*Burton F1	237	237	371	371	150	150	202	202	153	153	285	285	162	162	253	253
35	Atria F1*5	241	241	348	348	150	150	206	206	153	153	275	275	150	150	253	253
49	Autumn queen F1	70	70	325	325	150	150	202	202	145	145	275	275	162	162	273	273
58	8*Atria F1	241	241	374	374	30	30	208	208	60	60	275	275	155	155	253	253
75	Kranjsko okroglo*5	241	241	348	348	135	135	206	206	153	153	275	275	155	155	273	273
85	8*Atria F1	241	241	374	374	150	150	202	202	153	153	275	275	162	162	253	253
103	92*Atria F1	241	241	348	348	135	135	206	206	145	145	275	275	155	155	273	273
119	Kranjsko okroglo*(36*165)	237	237	325	325	150	150	206	206	153	153	275	275	155	155	253	253
123	Atria F1*7	237	237	325	325	150	150	196	196	148	148	275	275	150	150	253	253
139	Atria F1*6	241	241	325	325	150	150	206	206	143	143	275	275	155	155	273	273
164	Atria F1*7	241	241	325	325	150	150	202	202	148	148	275	275	162	162	253	253
168	Grandslam F1	70	70	374	374	150	150	210	210	153	153	275	275	162	162	253	253
171	Fieldwinner F1	241	241	325	325	135	135	210	210	145	145	275	275	155	155	253	253
176	Krautman F1	237	237	325	325	135	135	202	202	153	153	275	275	155	155	253	253
180	92*Kranjsko okroglo	241	241	374	374	150	150	196	196	153	153	275	275	162	162	273	273
190	Kranjsko okroglo*5	237	237	374	374	150	150	206	206	153	153	275	275	155	155	250	250
206	Kranjsko okroglo*7	237	237	374	374	150	150	196	196	145	145	275	275	155	155	253	253
247	Atria F1*165	237	237	325	325	150	150	208	208	153	153	275	275	150	150	253	253
253	5*Atria F1	241	241	325	325	135	135	206	206	143	143	275	275	150	150	253	253
260	Atria F1*7	237	237	325	325	150	150	208	208	153	153	275	275	150	150	273	273
264	8*Atria F1	241	241	374	374	150	150	202	202	148	148	275	275	162	162	253	253
273	278*Burton F1	241	241	371	371	135	135	202	202	153	153	285	285	162	162	253	253
292	5*Atria F1	241	241	325	325	135	135	202	202	143	143	275	275	155	155	253	253
304	Fieldwinner F1	70	70	348	348	150	150	208	208	145	145	275	275	155	155	273	273
306	Fieldwinner F1	241	241	348	348	150	150	210	210	145	145	275	275	155	155	273	273
344	Kranjsko okroglo*7	241	241	374	374	150	150	208	208	153	153	275	275	155	155	273	273
347	Kranjsko okroglo*165	237	237	368	368	150	150	202	202	148	148	275	275	155	155	253	253
349	278*Burton F1	237	237	371	371	135	135	196	196	153	153	285	285	162	162	253	253
