# Supplementary material for: Proposal of a New Hybrid Breeding Method Based on Genotyping, Inter-Pollination, Phenotyping and Paternity Testing of Selected Elite F1 Hybrids
Source: Front Plant Sci. 2019 Sep 18;10:1111. doi: 10.3389/fpls.2019.01111 (PMC6759491; doi:10.3389/fpls.2019.01111)
Supplement: Supplementary file 3 [file DataSheet_3.pdf]

Table S3: The origin of inbred lines used in creation of donor plants in Suppl. Table S2 and S6

Inbred lines	donor plant
2	667*885
4	712*Varaždinsko
5	667*Matsumo F1
6	667*Matsumo F1
7	667*885
8	667*Matsumo F1
92	667*885
165	667*885
278	R41*Varaždinsko
667	R1*Varaždinsko
712	R1*Varaždinsko
885	R41*Varaždinsko
R1	Hawke F1
R41	Hawke F1
