# Supplementary material for: Proposal of a New Hybrid Breeding Method Based on Genotyping, Inter-Pollination, Phenotyping and Paternity Testing of Selected Elite F1 Hybrids
Source: Front Plant Sci. 2019 Sep 18;10:1111. doi: 10.3389/fpls.2019.01111 (PMC6759491; doi:10.3389/fpls.2019.01111)
Supplement: Supplementary file 4 [file DataSheet_4.pdf]

**Table S4: The origin of varieties used as donor or in creation of donor plants in Suppl. Table S2, S3**

| Variety | Origin |
|---|---|
| Atria F1 | Semenarna Ljubljana |
| Autumn queen F1 | Takii Seed |
| Benelli F1 | Bejo Zaden |
| Burton F1 | Nickerson Zwaan |
| Fieldwinner F1 | Bejo Zaden |
| Grandslam F1 | Sakata |
| Krautman F1 | Bejo Zaden |
| Kranjsko okroglo | Slovene gene bank |
| Matsumo F1 | Bejo Zaden |
| Varaždinsko | Semenarna Ljubljana |
| Hawke F1 | English hybrid cultivar |

**and S6**
