# Supplementary material for: Proposal of a New Hybrid Breeding Method Based on Genotyping, Inter-Pollination, Phenotyping and Paternity Testing of Selected Elite F1 Hybrids
Source: Front Plant Sci. 2019 Sep 18;10:1111. doi: 10.3389/fpls.2019.01111 (PMC6759491; doi:10.3389/fpls.2019.01111)
Supplement: Supplementary file 5 [file DataSheet_5.pdf]

Table S5: Determination of male parent of F1 hybrids, LOD score and genotyping of F1 hybrids with eight molecular markers for cage experiment

	Determined male parent of F1			Genotyping of F1 hybrids with eight SSR markers															
			No. of selections with the same parent lines	locus 1		locus 2		locus 3		locus 4		locus 5		locus 6		locus 7		locus 8	
**No. (moth**	male p.	LOD																	
	11	5.80	1	241	241	325	371	135	150	196	202	145	153	275	275	162	162	273	273
1	28/281	5.17	1	241	241	371	374	135	150	196	202	153	153	275	275	162	162	253	273
	1	7.58	1	241	241	325	371	135	150	196	202	145	153	275	275	162	162	273	273
	79	6.27	2	241	241	325	325	135	150	196	202	145	148	275	275	155	162	253	273
11	192	8.67	1	241	241	325	348	150	150	196	206	145	153	275	275	155	162	253	273
	1	7.58	2	241	241	371	374	135	150	196	202	153	153	275	275	162	162	253	273
	59	5.51	1	241	241	325	374	150	150	196	202	148	153	275	275	155	162	253	253
	79	6.27	1	241	241	325	374	135	150	196	202	148	153	275	275	155	162	253	253
	272	7.71	3	237	241	371	374	135	150	196	196	153	153	275	275	162	162	253	253
28	311	9.46	1	241	-	374	374	150	150	196	210	145	153	275	275	162	162	253	253
40	275	9.51	3	237	241	325	325	135	135	202	208	153	-	275	285	155	162	253	273
	11	5.79	1	237	241	325	325	150	150	196	208	145	153	275	275	155	162	253	273
43	341	5.85	6	237	241	325	325	135	150	202	208	153	-	275	275	155	162	253	253
	1	7.57	1	241	241	325	371	135	150	202	206	153	153	275	275	155	162	253	273
	272	7.71	1	237	241	325	371	135	150	196	206	153	153	275	275	155	162	253	253
48	311	9.45	1	241	-	325	374	150	150	206	210	145	153	275	275	155	162	253	253
	43	6.79	1	237	241	325	374	150	150	196	208	145	153	275	275	155	162	253	273
	261	7.19	2	237	241	325	374	150	150	196	196	145	148	275	275	155	162	273	273
52	342	6.16	1	237	241	325	374	150	150	196	202	145	145	275	275	155	162	253	273
	52	6.63	1	237	241	371	374	150	150	196	202	145	153	275	275	162	162	273	273
65	121	7.90	4	237	241	325	371	150	150	202	202	143	153	275	275	150	162	253	273
	261	7.19	1	237	241	325	325	150	150	196	202	143	148	275	275	150	155	253	273
	311	9.45	2	241	-	325	374	150	150	202	210	143	145	275	275	150	162	253	253
121	341	5.85	1	241	241	325	325	135	150	202	202	143	-	275	275	150	162	253	253
	79	6.27	1	241	241	325	374	135	150	202	206	145	148	275	275	155	155	253	253
189	275	9.51	1	241	241	325	374	135	150	202	206	145	-	275	285	155	162	253	253
	11	5.80	3	241	241	325	348	150	150	196	206	145	153	275	275	155	162	253	273
	79	6.27	1	241	241	325	348	135	150	202	206	148	153	275	275	155	155	253	253
192	311	9.46	2	241	-	348	374	150	150	206	210	145	153	275	275	155	162	253	253
	11	5.79	3	237	241	325	325	150	150	196	196	145	153	275	275	155	162	253	273
210	311	9.45	1	237	-	325	374	150	150	196	210	145	153	275	275	155	162	253	253
	261	7.19	1	237	241	325	325	135	150	196	202	143	148	275	275	150	155	253	273
236	311	9.45	1	241	-	325	374	135	150	202	210	143	145	275	275	150	162	253	-
	79	6.27	1	241	241	325	374	135	150	202	208	148	153	275	275	155	162	253	253
	121	7.90	2	241	241	325	374	150	150	202	208	143	153	275	275	150	162	253	253
249	341	5.85	2	241	241	325	374	135	150	202	208	153	-	275	275	162	162	253	253

	79	6.27	1	237	241	325	325	135	150	196	202	148	148	275	275	155	155	253	273
261	341	5.85	19	237	241	325	325	135	150	196	202	148	-	275	275	155	162	253	273
	1	7.58	1	237	241	371	371	135	135	196	202	153	153	275	275	162	162	253	273
	121	7.90	1	237	241	325	371	135	150	196	202	143	153	275	275	150	162	253	253
272	236	8.66	1	237	241	325	371	135	135	196	202	143	153	275	275	150	162	253	253
274	121	7.90	1	241	241	325	371	150	150	196	202	143	153	275	275	150	162	253	273
275	121	7.90	1	241	241	325	325	135	150	202	202	143	-	275	285	150	162	253	253
276	249	6.39	1	241	241	374	374	135	150	202	208	153	153	275	275	155	162	253	253
	1	7.58	1	241	241	371	374	135	150	196	202	153	153	275	275	162	162	253	273
	11	5.80	5	241	241	325	374	150	150	196	196	145	153	275	275	162	162	253	273
	79	6.27	1	241	241	325	374	135	150	196	202	148	153	275	275	155	162	253	253
	275	9.51	1	241	241	325	374	135	150	196	202	153	-	275	285	162	162	253	253
281	311	9.46	2	241	-	374	374	150	150	196	210	145	153	275	275	162	162	253	253
	11	5.80	2	241	-	325	374	150	150	196	210	145	145	275	275	162	162	253	273
	28/281	5.17	2	241	-	374	374	150	150	196	210	145	153	275	275	162	162	253	253
	104	7.89	1	241	-	348	374	150	150	202	210	145	153	275	275	155	162	253	273
	121	7.90	4	241	-	325	374	150	150	202	210	143	145	275	275	150	162	253	253
	192	8.68	1	241	-	348	374	150	150	206	210	145	153	275	275	155	162	253	253
	210	5.58	1	237	-	325	374	150	150	196	210	145	153	275	275	155	162	253	253
	249	6.39	2	241	-	374	374	150	150	208	210	145	153	275	275	162	162	253	253
311	346	8.50	1	237	-	368	374	150	150	202	210	145	153	275	275	155	162	253	253
	43	6.79	3	237	241	325	325	135	150	202	208	153	-	275	275	155	162	253	253
	52	6.63	1	241	241	325	374	135	150	196	202	145	-	275	275	162	162	253	273
	65	7.62	1	237	241	325	371	135	150	202	202	153	-	275	275	162	162	253	273
	79	6.27	1	241	241	325	325	135	135	202	202	148	-	275	275	155	162	253	253
	249	6.39	1	241	241	325	374	135	150	202	208	153	-	275	275	162	162	253	253
341	261	7.19	9	237	241	325	325	135	150	196	202	148	-	275	275	155	162	253	273
342	11	5.79	1	237	241	325	325	150	150	196	202	145	145	275	275	155	162	253	273
