# Supplementary material for: Proposal of a New Hybrid Breeding Method Based on Genotyping, Inter-Pollination, Phenotyping and Paternity Testing of Selected Elite F1 Hybrids
Source: Front Plant Sci. 2019 Sep 18;10:1111. doi: 10.3389/fpls.2019.01111 (PMC6759491; doi:10.3389/fpls.2019.01111)
Supplement: Supplementary file 6 [file DataSheet_6.pdf]

Table S6: Determination of male parent of F1 hybrids LOD score and genotyping of F1 hybrids with eight molecular markers for open pollination experimen

	Determined male parent of F1			Genotyping of F1 hybrids with eight moleculars markers															
**DH plant No. (mother plant)**	male p.	LOD	No. of selection with the same parent lines	locus 1		locus 2		locus 3		locus 4		locus 5		locus 6		locus 7		locus 8	
	164	6.67	1	241	241	325	325	150	150	202	210	148	153	275	275	155	162	253	253
	260	6.92	1	237	241	325	325	150	150	208	210	153	153	275	275	150	155	253	273
7	349	11.30	1	237	241	325	371	135	150	196	210	153	153	275	285	155	162	253	253
	49	7.71	1	237	-	325	374	150	150	202	208	145	153	275	275	155	162	253	273
	176	5.88	2	237	237	325	374	135	150	202	208	153	153	275	275	155	155	253	253
15	247	6.29	1	237	237	325	374	150	150	208	208	153	153	275	275	150	155	253	253
	171	6.82	2	241	241	325	348	135	135	206	210	145	153	275	275	155	155	253	273
	306	8.01	1	241	241	348	348	135	150	206	210	145	153	275	275	155	155	273	273
75	349	11.30	1	237	241	348	371	135	135	196	206	153	153	275	285	155	162	253	273
	9	7.95	2	241	-	325	374	150	150	202	210	145	153	275	275	155	162	253	273
85	49	7.71	5	241	-	325	374	150	150	202	202	145	153	275	275	162	162	253	273
	176	5.88	1	237	241	325	348	135	135	202	206	145	153	275	275	155	155	253	273
103	260	6.92	1	237	241	325	348	135	150	206	208	145	153	275	275	150	155	273	273
	49	7.71	2	237	-	325	325	150	150	196	202	145	148	275	275	150	162	253	273
	176	5.88	1	237	237	325	325	135	150	196	202	148	153	275	275	150	155	253	253
	273	9.76	2	237	241	325	371	135	150	196	202	148	153	275	285	150	162	253	253
123	349	11.30	4	237	237	325	371	135	150	196	196	148	153	275	285	150	162	253	253
164	9	7.95	1	241	-	325	325	150	150	202	210	145	148	275	275	155	162	253	273
	26	9.47	1	237	-	371	374	150	150	202	210	153	153	275	285	162	162	253	253
	247	6.29	1	237	-	325	374	150	150	208	210	153	153	275	275	150	162	253	253
168	292	6.70	1	241	-	325	374	135	150	202	210	143	153	275	275	155	162	253	253
	35	7.66	1	237	241	325	348	135	150	202	206	153	153	275	275	150	155	253	253
176	247	6.29	1	237	237	325	325	135	150	202	208	153	153	275	275	150	155	253	253
	9	7.95	1	237	-	325	325	150	150	208	210	145	153	275	275	150	155	253	273
	168	8.15	2	237	-	325	374	150	150	208	210	153	153	275	275	150	162	253	253
	176	5.88	2	237	237	325	325	135	150	202	208	153	153	275	275	150	155	253	253
	253	8.31	1	237	241	325	325	135	150	206	208	143	153	275	275	150	150	253	253
247	273	9.76	1	237	241	325	371	135	150	202	208	153	153	275	285	150	162	253	253
	260	6.92	1	237	241	325	325	135	150	206	208	143	153	275	275	150	150	253	273
	304	8.91	1	241	-	325	348	135	150	206	208	143	145	275	275	150	155	253	273
	306	8.01	3	241	241	325	348	135	150	206	210	143	145	275	275	150	155	253	273
253	344	6.90	1	241	241	325	374	135	150	206	208	143	153	275	275	150	155	253	273
	273	9.75	1	237	241	325	371	135	150	202	208	153	153	275	285	150	162	253	273
260	292	6.70	3	237	241	325	325	135	150	202	208	143	153	275	275	150	155	253	273
	253	8.31	3	241	241	325	371	135	135	202	206	143	153	275	285	150	162	253	253
	260	6.92	1	237	241	325	371	135	150	202	208	153	153	275	285	150	162	253	273
273	292	6.70	1	241	241	325	371	135	135	202	202	143	153	275	285	155	162	253	253
	49	7.71	1	241	-	325	325	135	150	202	202	143	145	275	275	155	162	253	273
	164	6.67	1	241	241	325	325	135	150	202	202	143	148	275	275	155	162	253	253
	260	6.91	6	237	241	325	325	135	150	202	208	143	153	275	275	150	155	253	273
	306	8.01	2	241	241	325	348	135	150	202	210	143	145	275	275	155	155	253	273

292	349	11.30	1	237	241	325	371	135	135	196	202	143	153	275	285	155	162	253	253
	13	7.14	1	241	-	348	374	135	150	202	208	145	145	275	275	155	155	253	273
	247	6.29	2	237	-	325	348	150	150	208	208	145	153	275	275	150	155	253	273
304	264	7.68	3	241	-	348	374	150	150	202	208	145	148	275	275	155	162	253	273
	164	6.67	2	241	241	325	348	150	150	202	210	145	148	275	275	155	162	253	273
	253	8.31	2	241	241	325	348	135	150	206	210	143	145	275	275	150	155	253	273
306	292	6.70	1	241	241	325	348	135	150	202	210	143	145	275	275	155	155	253	273
344	306	8.01	3	241	241	348	374	150	150	208	210	145	153	275	275	155	155	273	273
	9	7.95	2	237	-	325	368	150	150	202	210	145	148	275	275	155	155	253	273
347	49	7.71	1	237	-	325	368	150	150	202	202	145	148	275	275	155	162	253	273
349	49	7.71	2	237	-	325	371	135	150	196	202	145	153	275	285	162	162	253	273
