# Supplementary material for: Proposal of a New Hybrid Breeding Method Based on Genotyping, Inter-Pollination, Phenotyping and Paternity Testing of Selected Elite F1 Hybrids
Source: Front Plant Sci. 2019 Sep 18;10:1111. doi: 10.3389/fpls.2019.01111 (PMC6759491; doi:10.3389/fpls.2019.01111)
Supplement: Supplementary file 7 [file DataSheet_7.pdf]

Table S7: Allele frequency analysis for eight loci for 282 genotypes used in cage and open pollination experiment

locus	k	N	HObs	HExp	PIC	HW	F(Null)
locus 1	3	282	0.496	0.558	0.482	NS	0.0590
locus 2	5	282	0.450	0.621	0.574	***	0.1528
locus 3	3	282	0.404	0.445	0.349	NS	0.0456
locus 4	5	282	0.621	0.752	0.713	***	0.0870
locus 5	5	282	0.631	0.726	0.686	***	0.0744
locus 6	2	282	0.089	0.110	0.104	ND	0.1080
locus 7	3	282	0.546	0.615	0.532	NS	0.0594
locus 8	3	282	0.429	0.446	0.349	NS	0.0172
